# Non-Cooperative Target Imaging and Parameter Estimation with Narrowband Radar Echoes

**DOI:** 10.3390/s16010125

**Published:** 2016-01-20

**Authors:** Chun-mao Yeh, Wei Zhou, Yao-bing Lu, Jian Yang

**Affiliations:** 1 Beijing Institute of Radio Measurement, Beijing 100039, China; danielgodman@163.com (C.Y.); luyaobing65@163.com (Y.L.); 2 Department of Electronic Engineering, Tsinghua University, Beijing 100084, China; yangjian_ee@mail.tsinghua.edu.cn

**Keywords:** narrowband radar, radar imaging, rotating object, rotation estimation

## Abstract

This study focuses on the rotating target imaging and parameter estimation with narrowband radar echoes, which is essential for radar target recognition. First, a two-dimensional (2D) imaging model with narrowband echoes is established in this paper, and two images of the target are formed on the velocity-acceleration plane at two neighboring coherent processing intervals (CPIs). Then, the rotating velocity (RV) is proposed to be estimated by utilizing the relationship between the positions of the scattering centers among two images. Finally, the target image is rescaled to the range-cross-range plane with the estimated rotational parameter. The validity of the proposed approach is confirmed using numerical simulations.

## 1. Introduction

High-resolution observation with coherent radar is always desired for precise target feature acquisition. The usually used high-resolution technique is inverse synthetic aperture radar (ISAR) [1], which transmits a wideband waveform for high-resolution in the range direction and coherently integrates target echoes from different aspects for high-resolution in the cross-range direction. The ISAR technique has been widely used for both civilian and military purposes [2,3]. However, the use of ISAR is often limited in real applications, especially for long-range detection and imaging situations. Firstly, the sensitivity of the wideband radar is worse than the narrowband radar according to the radar system design principle [4], and thus the wideband ISAR has much shorter range coverage than the narrowband radar. Secondly, the wideband radar tracking technique was not mature until now, and the ISAR system should transmit a narrowband waveform for target tracking. Thus, most ISAR systems transmit a wideband waveform and a narrowband waveform alternatively for both tracking and imaging purposes, resulting much lower data rate that may cause Doppler aliasing for rotating objects with large Doppler modulation bandwidths. Considering the above reasons, a narrowband waveform with high pulse repetition frequency (PRF) is usually preferred for long range target tracking and feature extraction.

With highly-repeated narrowband echoes, high-resolution cross-range profiles (HRCRP) may be obtained [5,6] after effective translation motion compensation (TMC) [7], and the HRCRP series forms the usually-called time frequency representation which is often exploited to demonstrate the micro-motion of the target [8]. What is more, two-dimensional (2D), and even three-dimensional (3D), images may also be obtained based on the narrowband signal processing methods [9,10,11,12]. However, all these methods are based on the assumption that the target’s rotating velocity (RV) is a known parameter, which is usually invalid for a non-cooperative object.

For wideband ISAR imaging, a number of methods have been proposed for the RV estimation, and these methods may be roughly categorized into two classes. The first is to search the RV which provides the best image focusing quality [13,14,15]. The other is to estimate the RV by exploiting the target’s pose difference on an RD image series [16,17,18]. For a narrowband imaging scene, the target’s RV can be estimated by making use of the radar echoes from T/R-R bistatic radar [19,20], which is not suitable for monostatic radar applications. For monostatic narrowband radar, the RV can be estimated by taking the autocorrelation of the received signal [10]. However, this method only works when a relatively large processing dwell is allowed. When the radar tracks the target for less than one rotating period, the estimated result will be invalid.

In this paper, the 2D radar imaging principle with narrowband echoes is reviewed, and the corresponding linear relationship is established to map the scattering centers from the 2D physical plane to the velocity-acceleration imaging plane. Then, the received echoes are divided into two neighboring coherent processing intervals (CPIs), and two images are formed on the velocity-acceleration plane through adaptive joint time-frequency (AJTF) techniques [21]. Then, both the image correlation method and the analytical method are proposed for the RV estimation. Finally, the target image may be rescaled to the range-cross-range plane with the estimated RV, which will greatly benefit the following target recognition. With the proposed methods, effective RV may be estimated within a time interval of only a fraction of the rotating period.

The reminder of this paper is organized as follows. The narrowband imaging model of a rotating objects is described in Section 2. Then, the rotation estimation methods are proposed in Section 3. Section 4 presents the numerical simulations, from which some conclusions are drawn in Section 5.

## 2. Narrowband Imaging Model of Rotating Objects

### 2.1. Narrowband Signal Model

For an object with multiple scattering centers, the phase history is commonly formulated as [5]:

(1) $$s\left(t_{m}\right)=\sum_{k}\sigma_{k}\exp\left(-j4\pi \frac{f_{c}}{c}r_{k}\left(t_{m}\right)\right)$$

where $\sigma_{k}$ is the complex amplitude of the *k*th scattering center, $f_{c}$ is the carrier frequency, c is the velocity of light, $t_{m}=mT_{r}$ is the pulse sampling time, m is the pulse number, $T_{r}$ is the pulse repetition interval, and $r_{k}\left(t_{m}\right)$ is the time-varied slant range of the *k*th scattering center.

**Figure 1:**
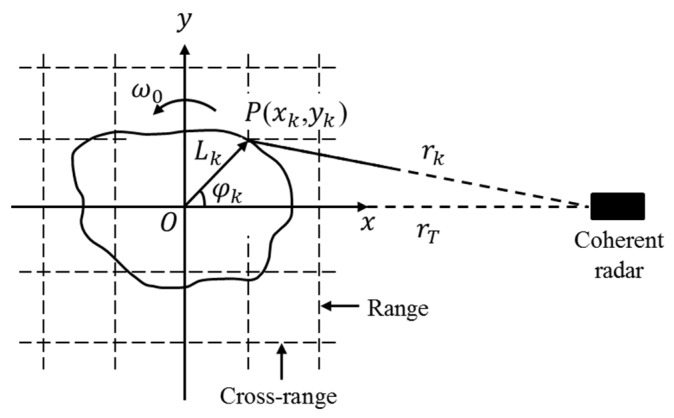
Rotating object geometry.

For a planar rotating object under far-field conditions, as shown in Figure 1, the range may usually be modeled as:

(2) $$\begin{array}{ccc} r_{k}\left(t_{m}\right) & \approx & r_{T}\left(t_{m}\right)-L_{k}\cos\left(\theta_{A}\left(t_{m}\right)+\varphi_{k}\right)\\ & = & r_{T}\left(t_{m}\right)-\left[x_{k}\cos\theta_{A}\left(t_{m}\right)-y_{k}\sin\theta_{A}\left(t_{m}\right)\right] \end{array}$$

where $r_{T}\left(t_{m}\right)$ denotes the translational motion between the radar and the rotating center of the object, $L_{k}$ denotes the rotating radius of the *k*th scattering center, $\theta_{A}\left(t_{m}\right)$ denotes the rotated aspect angle, $\varphi_{k}$ denotes the initial phase of the *k*th scattering center, $x_{k}=L_{k}\cos\varphi_{k}$, $y_{k}=L_{k}\sin\varphi_{k}$, and $\left(x_{k},y_{k}\right)$ denotes the position of the *k*th scattering center in the local coordinate system.

For a uniformly-rotating object, $\theta_{A}\left(t_{m}\right)=\omega_{o}t_{m}$, where $\omega_{o}$ is the RV. Thus, the slant range of the *k*th scattering center may be approximated with a second-order Taylor’s expansion as follows [15]:

(3) $$r_{k}\left(t_{m}\right)\approx \left(r_{T}\left(t_{m}\right)-x_{k}\right)+y_{k}\omega_{o}t_{m}+0.5x_{k}\omega_{o}^{2}t_{m}^{2}$$

Combined with Equation (2), the Doppler frequency of the *k*th scattering center is:

(4) $$f_{Dk}\left(t_{m}\right)=\frac{2}{\lambda }v_{k}\left(t_{m}\right)\approx \frac{2}{\lambda }\left(v_{T}\left(t_{m}\right)+y_{k}\omega_{o}+x_{k}\omega_{o}^{2}t_{m}\right)$$

where $\lambda =c/f_{c}$ is the wavelength, $v_{k}={\dot{r}}_{k}\left(t_{m}\right)$ is the first order derivative of the slant range $r_{k}\left(t_{m}\right)$, and $v_{T}\left(t_{m}\right)$ is the translational velocity of the rotating center.

From Equation (4), the Doppler frequency of the *k*th scattering center is linearly varied, and the Doppler modulation rate is:

(5) $$\gamma_{Dk}=\frac{2}{\lambda }\alpha_{k}\left(t_{m}\right)\approx \frac{2}{\lambda }\left(\alpha_{T}\left(t_{m}\right)+x_{k}\omega_{o}^{2}\right)$$

where $\alpha_{k}\left(t_{m}\right)={\dot{v}}_{k}\left(t_{m}\right)$, and $\alpha_{T}\left(t_{m}\right)$ is the translational acceleration of the scattering center.

Without the influence of the translational motion, the central Doppler frequency $f_{Dk}$ and the Doppler frequency modulation rate $\gamma_{Dk}$ are related to the cross-range position $y_{k}$ and range position $x_{k}$ of the *k*th scattering center, respectively as:

(6) $$\begin{array}{cc} f_{Dk}\approx \frac{2\omega_{o}}{\lambda }y_{k}, & \gamma_{Dk}\approx \frac{2\omega_{o}^{2}}{\lambda }x_{k} \end{array}$$

From the above analysis, it is concluded that the narrowband echoes of a rotating object may be modeled as a multi-component polynomial phase signal. The phase coefficients are related to the wavelength, the position of each scattering center, and the RV of the object. Usually, the time frequency analysis (TFA) technique is exploited to demonstrate the rotational and scattering characteristics of the target after effective TMC, and the Doppler bandwidth is related to the cross-range size and the RV of the object. In real applications, the cross-range size information of the target should not be available without $\omega_{o}$, which is usually unknown for a non-cooperative object.

What is more, the TFA method may only provide the cross-range information of a scattering center. In fact, with the central Doppler frequency and Doppler frequency modulation rate, a scattering center may also be demonstrated on the range and cross-range domain as illustrated in Equation (6). When the 2D distribution of the scattering centers is provided, more accurate shape and size information of the target should be deduced, and thus facilitate the radar target recognition afterwards.

### 2.2. 2D Imaging for Uniformly Rotating Objects

For a planar rotating object with constant RV, the radial velocity and acceleration of the *k*th scattering center may be more precisely represented according to Equation (2):

(7) $$v_{k}\left(t_{m}\right)=v_{T}\left(t_{m}\right)+\omega_{o}\left[x_{k}\sin\left(\omega_{o}t_{m}\right)+y_{k}\cos\left(\omega_{o}t_{m}\right)\right]$$

(8) $$\alpha_{k}\left(t_{m}\right)=\alpha_{T}\left(t_{m}\right)+\omega_{o}^{2}\left[x_{k}\cos\left(\omega_{o}t_{m}\right)-y_{k}\sin\left(\omega_{o}t_{m}\right)\right]$$

Combining Equations (7) and (8), a linear mapping relationship may be established between the position of scattering centers and their corresponding radial velocity and acceleration:

(9) $$\left[\begin{array}{c} \alpha_{k}\left(t_{m}\right)\\ v_{k}\left(t_{m}\right) \end{array}\right]=\left[\begin{array}{c} \alpha_{T}\left(t_{m}\right)\\ v_{T}\left(t_{m}\right) \end{array}\right]+CR\left(t_{m}\right)\left[\begin{array}{c} x_{k}\\ y_{k} \end{array}\right]$$

where C is the scaling matrix determined by the RV, $R\left(t_{m}\right)$ is the instantaneous aspect matrix:

(10) $$C=\left[\begin{array}{cc} \omega_{o}^{2} & \\ & \omega_{o} \end{array}\right]$$

(11) $$R\left(t_{m}\right)=\left[\begin{array}{cc} \cos\left(\omega_{o}t_{m}\right) & -\sin\left(\omega_{o}t_{m}\right)\\ \sin\left(\omega_{o}t_{m}\right) & \cos\left(\omega_{o}t_{m}\right) \end{array}\right]$$

Suppose a total number of *K* scattering centers are extracted; then an observation matrix $G\left(t_{m}\right)$ composed of the centralized radial velocity and acceleration may be obtained:

(12) $$G\left(t_{m}\right)=\left[\begin{array}{cccc} {\bar{\alpha }}_{1}\left(t_{m}\right) & {\bar{\alpha }}_{2}\left(t_{m}\right) & \cdots & {\bar{\alpha }}_{K}\left(t_{m}\right)\\ {\bar{v}}_{1}\left(t_{m}\right) & {\bar{v}}_{2}\left(t_{m}\right) & \cdots & {\bar{v}}_{K}\left(t_{m}\right) \end{array}\right]$$

where ${\bar{v}}_{k}\left(t_{m}\right)$ is the centralized radial velocity, and ${\bar{\alpha }}_{k}\left(t_{m}\right)$ is the centralized radial acceleration:

(13) $$\begin{array}{cc} {\bar{v}}_{k}\left(t_{m}\right)=v_{k}\left(t_{m}\right)-\frac{1}{K}\sum_{n=1}^{K}v_{n}\left(t_{m}\right), & k=1,\cdots ,K \end{array}$$

(14) $$\begin{array}{cc} {\bar{\alpha }}_{k}\left(t_{m}\right)=\alpha_{k}\left(t_{m}\right)-\frac{1}{K}\sum_{n=1}^{K}\alpha_{n}\left(t_{m}\right), & k=1,\cdots ,K \end{array}$$

According to Equation (12), a centralized form of the linear mapping relationship is expressed as:

(15) $$G\left(t_{m}\right)=CR\left(t_{m}\right)P$$

where P is the centralized position matrix of scattering centers:

(16) $$P=\left[\begin{array}{cccc} {\bar{x}}_{1} & {\bar{x}}_{2} & \cdots & {\bar{x}}_{K}\\ {\bar{y}}_{1} & {\bar{y}}_{2} & \cdots & {\bar{y}}_{K} \end{array}\right]$$

(17) $$\begin{array}{ccc} {\bar{x}}_{k}=x_{k}-\frac{1}{K}\sum_{n=1}^{K}x_{n}, & {\bar{y}}_{k}=y_{k}-\frac{1}{K}\sum_{n=1}^{K}y_{n} & k=1,\cdots ,K \end{array}.$$

Therefore, with narrowband echoes, a 2D image may be formed on the velocity-acceleration plane for a planar rotating object, and the pose of the target varies with the aspect angle. For real applications, the pose of the target may vary from one CPI to another. This may be exploited for target rotation estimation and, thus, help target feature extraction from narrow-band echoes.

## 3. Rotation Motion Estimation

In this section, a rotation estimation scheme with narrowband echoes are proposed, and two implementation methods are proposed. With the estimated RV, the images of the rotating target may be size-scaled for better target feature extraction.

### 3.1. Analytic Rotation Estimation

When the radial velocity and acceleration of multiple prominent scattering centers are extracted from two neighboring CPIs, the observation matrices may be expressed as:

(18) $$G\left(t_{c1}\right)=CR\left(t_{c1}\right)P$$

(19) $$G\left(t_{c2}\right)=CR\left(t_{c2}\right)P$$

where $G\left(t_{c1}\right)$ and $G\left(t_{c2}\right)$ are the observation matrices obtained from the first CPI and second CPI, respectively, $t_{c1}$ is the central time of the first CPI, $t_{c2}$, and is the central time of the second CPI. 

Combing Equations (18) and (19), we can obtain:

(20) $$G\left(t_{c2}\right)=H\left(\theta_{d}\right)G\left(t_{c1}\right)$$

where $H\left(\theta_{d}\right)$ is the generalized rotation matrix:

(21) $$\begin{array}{ccc} H\left(\theta_{d}\right) & = & CR\left(t_{c2}\right)R^{-1}\left(t_{c1}\right)C^{-1}=\left[\begin{array}{cc} h_{1} & h_{2}\\ h_{3} & h_{4} \end{array}\right]\\ & = & \left[\begin{array}{cc} \cos\theta_{d} & -\omega_{o}\sin\theta_{d}\\ \left(1/\omega_{o}\right)\sin\theta_{d} & \cos\theta_{d} \end{array}\right] \end{array}$$

where $\theta_{d}=\omega_{o}\left(t_{c2}-t_{c1}\right)$ is the aspect angle difference of the target between neighboring CPIs. The aspect angle difference may be extracted from the generalized rotation matrix as:

(22) $$\theta_{d}=\frac{1}{2}\mathrm{acos}\left(h_{1}h_{4}+h_{2}h_{3}\right)$$

With the above analysis, if multiple scattering centers are extracted and correctly associated between neighboring CPIs, the generalized rotation matrix may be estimated as:

(23) $$\hat{H}\left(\theta_{d}\right)=\left(\hat{G}\left(t_{c2}\right){\hat{G}}^{T}\left(t_{c1}\right)\right){\left(\hat{G}\left(t_{c1}\right){\hat{G}}^{T}\left(t_{c1}\right)\right)}^{-1}$$

where $\hat{G}\left(t_{c1}\right)$ and $\hat{G}\left(t_{c2}\right)$ are the estimated observation matrices.

It should be pointed that at least three scattering centers should be extracted from both of the neighboring CPIs for the above analytical rotation estimation, and these scattering centers should not lie on a line for effective estimation.

### 3.2. Rotation Estimation by Image Rotation Correlation

With the extracted radial velocity and acceleration of scattering centers, a 2D image of the target may be reconstructed as:

(24) $$f\left(v,\alpha \right)=\sum_{k=1}^{K}\zeta_{k}\mathrm{sinc}\left(\frac{1}{\rho_{v}}\left(v-v_{k}\right)\right)\mathrm{sinc}\left(\frac{1}{\rho_{\alpha }}\left(\alpha -\alpha_{k}\right)\right)$$

where $\rho_{v}=\lambda /\left(2T_{obs}\right)$ is the velocity resolution, $\rho_{\alpha }=\lambda /\left(2T_{obs}^{2}\right)$ is the acceleration resolution, and $T_{obs}$ is the time duration of each CPI.

With the scattering centers extracted and effectively associated from the neighboring CPIs, two images should be obtained. According to the linear mapping relation in Equation (20), the image obtained from the second CPI should have the same target pose as the image obtained from the first CPI after image rotation with the right rotating angle. Thus, the RV may be estimated by image rotational correlation. The similarity between two images is designed as:

(25) $$S\left(\tilde{\omega }\right)=\frac{\iint \left|f\left(v,\alpha |t_{m2}\right)\right|\left|\tilde{f}\left(v,\alpha |t_{m1}\right)\right|dvd\alpha }{\sqrt{\iint {\left|f\left(v,\alpha |t_{m2}\right)\right|}^{2}dvd\alpha }\sqrt{\iint {\left|\tilde{f}\left(v,\alpha |t_{m1}\right)\right|}^{2}dvd\alpha }}$$

where $f\left(v,\alpha |t_{m2}\right)$ is the image formed from the second CPI, and $\tilde{f}\left(v,\alpha |t_{m1}\right)$ is the rotated version of the image $f\left(v,\alpha |t_{m1}\right)$ which is formed from the first CPI according to the given RV of $\tilde{\omega }$.

The time-consuming 2D image interpolation is required for the calculation of $\tilde{f}\left(v,\alpha |t_{m1}\right)$. Here, a more efficient three-step processing scheme is proposed to rotate the image by factorizing the rotating matrix as:

(26) $$H\left(\theta_{d}\right)=A_{\alpha }A_{v}A_{\alpha }$$

where $A_{\alpha }$ denotes image shearing in the acceleration direction, and $A_{v}$ denotes image shearing in the velocity direction.

(27) $$\begin{array}{cc} A_{\alpha }=\left[\begin{array}{cc} 1 & -\omega_{o}\tan\left(\theta_{d}/2\right)\\ 0 & 1 \end{array}\right], & A_{v}=\left[\begin{array}{cc} 1 & 0\\ \left(1/\omega_{o}\right)\sin\theta_{d} & 1 \end{array}\right] \end{array}$$

Thus, the whole image rotation transform can be decomposed into a sequence of 1D signal translations that may be implemented by Fast Fourier Transform (FFT).

Obviously, $S\left(\tilde{\omega }\right)$ is a function for the similarity measurement between two images, and it is supposed that this function reaches its maximum only with the right RV, *i.e.*,

(28) $${\hat{\omega }}_{o}=\underset{\tilde{\omega }}{\max}S\left(\tilde{\omega }\right)$$

Consequently, with the estimated RV, the image may be size scaled in the range-cross-range domain according to Equation (6).

### 3.3. Scattering Center Extraction and Association

The scattering center extraction and association is a necessary process for both the above RV estimation methods. The radial velocity and acceleration of scattering centers may be extracted through the AJTF technique proposed in [21]. The high-order phase coefficients are extracted by coherently accumulating a number of echoes with a CLEAN technique, and the performance for parameter estimation may follow the same principle as in [22]. The main procedure is listed below.

(1) The AJTF method is initialized as p=0, $s_{p}\left(t_{m}\right)=s\left(t_{m}\right)$.
(2) The basis function in our method is constructed as:
  (29) $$h_{p}\left(t_{m}|v_{p},\alpha_{p}\right)=\exp\left(-j4\pi \frac{f_{c}}{c}\left(v_{p}t_{m}+\alpha_{p}t_{m}^{2}\right)\right)$$
  where $\left(v_{p},\alpha_{p}\right)$ is the radial velocity and acceleration which we want to estimate.
(3) The projection value is then defined as:
  (30) $$B_{p}\left(v_{p},\alpha_{p}\right)=\left|\int s\left(t_{m}\right)h_{p}^{*}\left(t_{m}|v_{p},\alpha_{p}\right)dt_{m}\right|$$
  and the parameters are estimated by:
  (31) $$\left({\hat{v}}_{p},{\hat{\alpha }}_{p}\right)=\underset{\left(v_{p},\alpha_{p}\right)}{\max}B_{p}\left(v_{p},\alpha_{p}\right)$$
(4) The component belonging to the pth scatter is removed from the signal, as:
  (32) $$s_{p+1}\left(t_{m}\right)=s_{p}\left(t_{m}\right)-B_{p}\left({\hat{v}}_{p},{\hat{\alpha }}_{p}\right)h_{p}\left(t_{m}|{\hat{v}}_{p},{\hat{\alpha }}_{p}\right)$$

Then the processing from step (2) to step (4) is repeated with p=p+1 until the energy of the residual signal is lower than the threshold. In addition, when the estimated projection value is smaller than the preset threshold, the iterative procedures also terminate. When all the scatters have been extracted, the scatters are then mapped to the velocity-acceleration plane according to Equations (4) and (5).

If the image formed at the first CPI is $I_{1}$, and the image formed at the first CPI is $I_{2}$, then, the multiple scattering centers on $I_{1}$ and $I_{2}$ may be associated by using the nearest neighboring method.

For the kth scattering center, the estimated position on $I_{1}$ is $\left[{\hat{v}}_{k}\left(t_{c1}\right),{\hat{\alpha }}_{k}\left(t_{c1}\right)\right]$. Then, its radial velocity at the second CPI is supposed to be:

(33) $${{\hat{v}}^{\prime }}_{k}={\hat{v}}_{k}\left(t_{c1}\right)+{\hat{\alpha }}_{k}\left(t_{c1}\right)\left(t_{c2}-t_{c1}\right)$$

The distance between the kth scattering center on $I_{1}$ and the nth scattering center on $I_{2}$ is then defined by:

(34) $$d_{nk}=\left|{{\hat{v}}^{\prime }}_{k}-{\hat{v}}_{n}\left(t_{c2}\right)\right|$$

Suppose:

(35) $$\hat{n}=\underset{n}{\min}d_{nk}$$

then, the kth scattering center on $I_{1}$ and the $\hat{n}\mathrm{th}$ scattering center on $I_{2}$ are associated with each other.

From the above analysis, we can see that at least three scattering centers should be correctly associated from the neighboring CPIs for the analytical rotation estimation method, while just the strongest scattering center should be associated for the image rotation correlation method.

## 4. Experimental Results

In this section, rotation estimation experiments with both the analytical method and the image correlation method are provided to demonstrate the effectiveness of the proposed methods. In all of the following experiments, the radar system parameters and the translational motion parameters of the targets keep the same. 

The radar works in the X-band (10 GHz) and transmits coherent linear frequency modulated (LFM) waveform. The pulse duration is 100 µs, the modulated bandwidth is 10 MHz, and the pulse repetition frequency is 500 Hz. The echoes are I/Q sampled with a sampling frequency of 20 MHz. A total number of 1024 pulses are sampled, corresponding to a time interval of 2.048 s.

The initial radial range of the rotating center is 370 km, and the initial translational radial velocity is set to be 1.5 km/s, the second and third order coefficients of the translational motion are set to be 10 m/s^2^ and 0.5 m/s^3^, respectively.

### 4.1. Numerical Experiments with Isolated Scattering Centers

A planar rotating object with isolated scattering centers located at (0, 0) m, (4, 0) m, (0, 4) m, (−4, 0) m, and (0, −4) m is exploited to demonstrate the effectiveness of the proposed rotation estimation methods. The target rotates with a constant velocity of 0.4 rad/s.

**Figure 2:**
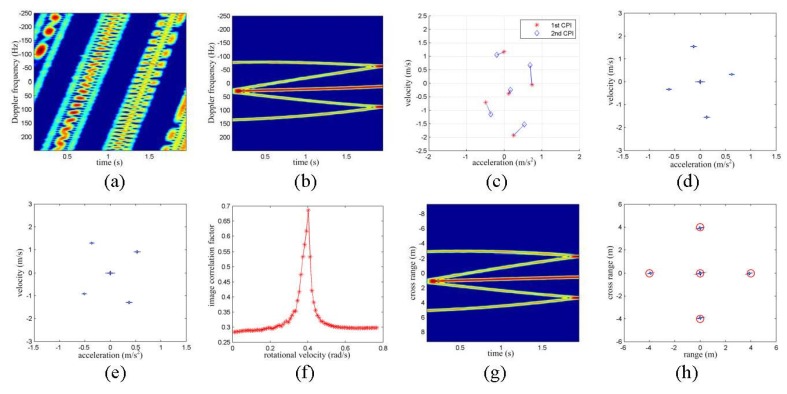
Numerical results with isolated scattering centers. (**a**) TFR of the received echoes; (**b**) TFR after TMC; (**c**) scattering center extraction and association result; (**d**) 2D image with first CPI; (**e**) 2D image with second CPI; (**f**) correlation coefficients with different RV; (**g**) cross-range scaled HRCRPs; and (**h**) range and cross-range scaled image with narrowband echoes.

The time frequency representation (TFR) of the target’s echoes is given in Figure 2a. It is shown that with the impact of the translational motion, little information should be extracted. The translational motion of the target is estimated and compensated with the method given in [7], and then the TFR of the target is given in Figure 2b. It is shown that there are five prominent scattering centers on the target, and the Doppler frequency of each scattering center varies with a different rate. The 1024 pulses are equally divided into two adjacent CPIs, *i.e.*, the first CPI covers the 1~512 pulses and the second CPI covers the pulses from 513 to 1024. After that, the AJTF technique is exploited to extract the amplitude, central frequency and frequency modulation rate of every scattering center from each CPI. The scattering centers are associated with the nearest neighboring method. The extraction and association result of the scattering centers is given in Figure 2c, in which the velocity and acceleration correspond to the central frequency and frequency modulation rate of the scattering centers, respectively. Finally, the analytical rotation estimation method is adopted to provide the estimation of the rotational velocity. The rotational velocity is estimated to be 0.3912 rad/s. According to Equation (36), the locations of the scatter centers are estimated to be (0, 0) m, (4.106, 0.020) m, (−0.085, 4.047) m, (0.039, −4.036) m, and (−4.151, −0.008) m, respectively:

(36) $$P=R^{-1}\left(t_{m}\right)C^{-1}G\left(t_{m}\right)$$

Additionally, with the extracted information of scattering centers, two 2D images are generated on the acceleration-velocity plane, such as in Figure 2d,e. The rotational velocity of the target is, thus, searched with the image rotational correlation method, and the curve in Figure 2f shows how the correlation coefficient varies with the rotational velocity. Thus, the rotational velocity is estimated to be 0.403 rad/s. With the estimation result, the TFR of the received echoes may be cross-range scaled as in Figure 2g, and the 2D image may be scaled in the range-cross-range plane as in Figure 2h. Obviously, with the effective rotational motion estimation, the shape and the size of a target may be better presented, which may facilitate the following target recognition.

### 4.2. Experiments with RCS Simulation

In the above experiments, each scattering center has constant amplitude during the entire CPI. In real applications, the echoes are noisy. The amplitude of the prominent scattering centers may vary and, thus, challenges the narrowband parameter estimation process.

**Figure 3:**
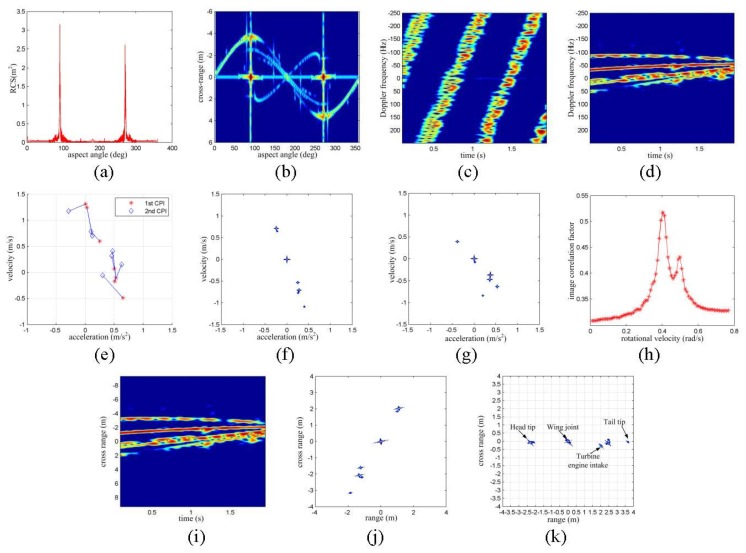
Experiments with RCS Simulation. (**a**) RCS variation with aspect angle; (**b**) cross-range scaled HRCRPs of the ALCM; (**c**) TFR without TMC; (**d**) TFR with TMC; (**e**) extraction and association of scattering centers; (**f**) 2D image with first CPI; (**g**) 2D image with second CPI; (**h**) correlation coefficients with different RV; (**i**) cross-range scaled HRCRPs; (**j**) range and cross-range scaled image; and (**k**) target image rotated to the horizontal direction.

In this experiment, the electromagnetic data of an air-launched cruise missile (ALCM) provided by [8] is exploited to generate narrowband radar echoes. The simulated radar system provides the signal to noise ratio (SNR) of 15 dB for an object with the radar cross section (RCS) of 1 m^2^ at the range of 600 km. The simulated SNR of each echo varies with the range and the temporary RCS of the target, as shown in Figure 3a. A total of 8192 electromagnetic samples are recorded which covers the 360° aspect angle. The length of the cruise missile is 6.4 m and its wingspan is about 3.4 m. The size-scaled cross-range profiles generated by the RCS data are presented in Figure 3b. The maximum span in the cross-range direction occurs at the aspect angle of about 110°, which shows the maximum length of the target is about 6 m. Additionally, there are three or four prominent scattering centers in the aspect angle interval between 110° and 250° as shown in Figure 3b. 

In the experiment, the initial aspect angle is set to be 120°, and the target rotates uniformly at a rate of 0.4 rad/s. A total number of 1024 echoes are collected in the 2.048 s interval. The initial TFR of the received echoes is given in Figure 3c, and little information is presented because of impact of the translational motion. After TMC, at least four prominent scattering centers are presented in the time-frequency plane as in Figure 3d. However, little information about the size and micro-motion of the target should be seen.

Following the same processing scheme in the above section, the amplitude, central frequency and frequency modulation rate of seven prominent scattering centers are extracted with the AJTF technique. The scattering centers are associated and presented on the acceleration and velocity plane as in Figure 3e. After that, the rotational motion of the target is estimated as 0.5653 rad/s, which presents prominent bias from the true value of the assumed rotating velocity. This may reason from the fact that the prominent scattering centers of the ALCM lie around a line and, thus, makes the analytical method invalid according to the former analysis.

With the extracted information of the scattering centers, the target may be presented in the acceleration-velocity plane as in Figure 3f,g. Then, the rotational correlation method is exploited to estimate the rotational velocity of the target. The rotational velocity is estimated to be 0.4029 rad/s according to the maximum correlation coefficient as in Figure 3h. Obviously, the image rotation correlation method provides better rotating velocity estimation in spite of the line-like target shape.

With the estimated RV, the TFR of the ALCM may be cross-range scaled as in Figure 3i, which shows the initial size of the target is about 5 m in the cross-range direction, and the cross-range size gets smaller as the target rotates toward the aspect angle of 180°. Additionally, the range and cross-range size scaled image of the target is given in Figure 3j, which may give more precise size and shape information about the target. According to the experimental assumptions, the size scaled image is 120° rotated as shown in Figure 3k. Refer to [8], the structure of the ALCM may be reflected by the position of the prominent scattering centers in the well scaled range and cross-range domain.

### 4.3. Experiments for Accelerated Rotating Objects

In this part, the impact of the rotational acceleration on the RV estimation will be examined.

**Figure 4:**
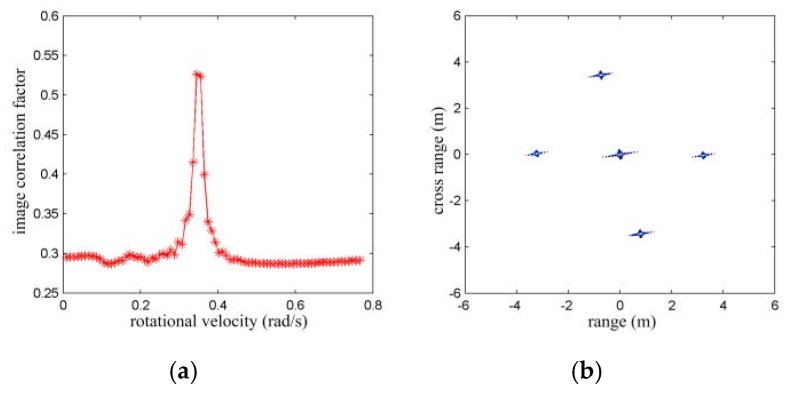
RV estimation for accelerated rotating object with isolated scattering model. (**a**) Correlation coefficients with different RV; and (**b**) range and cross-range scaled image with the estimated RV by image rotation correlation method.

In the first case, a target formed by five isolated prominent scattering centers as in Figure 2a is used. The initial RV and the rotating acceleration are set to be 0.3 rad/s and 0.02 rad/s^2^, respectively. The above parameter estimation scheme is applied. With the analytical method, the rotating velocity is estimated as 0.1242 rad/s and with the image rotation correlation method, the rotating velocity is estimated as 0.3454 rad/s as shown in Figure 4a. The image rotation correlation method provides remarkably better rotating velocity estimation than the analytical method. With the estimated rotating velocity of 0.3454 rad/s, the image may be size scaled in the range and cross-range domain as in Figure 4b. The locations of scattering centers are (0, 0) m, (3.238, −0.042) m, (−0.720, 3.436) m, (−3.238, 0.042) m, and (0.780, −3.436) m. From the imaging result, one may observe target deformation and size bias.

**Figure 5:**
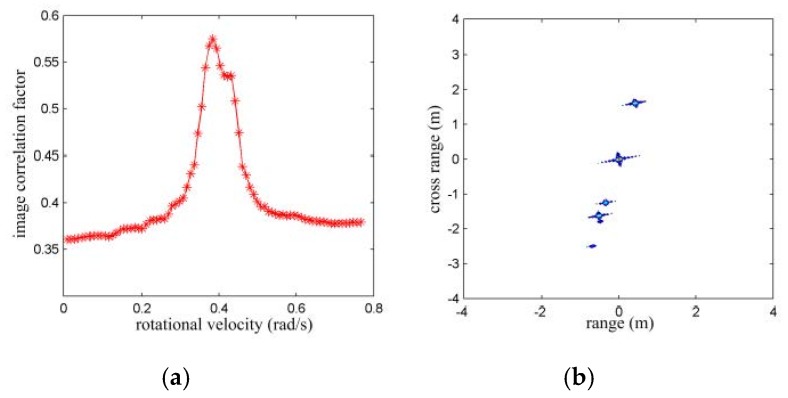
RV estimation with accelerated rotating ALCM. (**a**) Correlation coefficients with different RV; and (**b**) range and cross-range scaled image with the estimated RV by image rotation correlation method.

In the second case, the electromagnetic data of the above ALCM is used. The initial aspect angle is set to be 120°, and the rotating velocity and acceleration are also set to be 0.3 rad/s and 0.02 rad/s^2^, respectively. No valid rotating velocity estimation result can be provided with the analytical estimation method, while a rotating velocity estimation of 0.3837 rad/s is provided by the image rotation correlation method, as shown in Figure 5a. With the estimated rotating velocity, the range and cross-range scaled imaging result of the ALCM is given in Figure 5b. In comparison to the imaging result in Figure 3j, the size of the ALCM is smaller than its true value with this accelerated rotation.

### 4.4. Some Analyses

From the above simulations, we can see that both the proposed RV estimation methods work well when the target rotates with a constant RV. However, there are still some main differences between them. First, the analytical method has a much lower computation load. With the attracted scattering centers, the association of the scattering centers and the RV estimation can be done with high efficiency. If there are K scatterers extracted from the received echoes, then the association of the scatterers needs about $K^{2}$ times calculation of the distance between each scatterer by Equation (34). In this case, the estimated observation matrices $\hat{G}\left(t_{c1}\right)$ and $\hat{G}\left(t_{c2}\right)$ have two rows and K columns, and the calculation of the $\hat{H}\left(\theta_{d}\right)$ by Equation (23) has a computation complexity of about O(8K). Therefore, the total computation complexity of the analytical method is about $O(K^{2}+8K)$. While the image correlation method needs to rotate the target image and search the image correlation coefficient with different RV, resulting a relative heavier computation load. In the proposed method, the 2D image rotating procedure is decomposed into a sequence of 1-D image translations that may be implemented by FFT, as shown in Equations (26) and (27). If the target image has $N_{x}$ acceleration cells and $N_{y}$ velocity cells, the computational load to rotate this image is mainly composed of $4N_{y}$ times of $N_{x}$-point FFTs for signal shifting in the acceleration direction and 2$N_{x}$ times of $N_{y}$-point FFTs for signal shifting in the velocity direction. Thus, such 2D image rotation requires $N_{x}N_{y}\left(2\log_{2}N_{x}+\log_{2}N_{y}\right)$ complex multiplications. If the maximum size of the target on the range and cross-range plane is L, then we can conclude from Equations (7) and (8) that the minimum number of acceleration and velocity cells will be:

(37) $$\left\{ \begin{array}{l} N_{x}=\frac{\underset{k}{\max}\left|\alpha_{k}\right|}{\rho_{\alpha }}=\frac{2\omega_{o}^{2}LT_{obs}^{2}}{\lambda }\\ N_{y}=\frac{\underset{k}{\max}\left|v_{k}\right|}{\rho_{v}}=\frac{2\omega_{o}LT_{obs}}{\lambda } \end{array}\right.$$

Since the 2D image correlation also needs $N_{x}N_{y}$ complex multiplications, the total computational load for the image correlation method will be $N_{\omega }N_{x}N_{y}\left(2\log_{2}N_{x}+\log_{2}N_{y}+1\right)$, where $N_{\omega }$ is the number of searched RVs. Therefore, the computation complexity of the image correlation method is much larger than the analytical method.

Second, the performance of the analytical method degrades when the attracted scattering centers lie around a line. According to Equations (12)–(14), the rank of $\hat{G}\left(t_{c1}\right)$ in Equation (23) will be one when all the scattering centers lie on a line, and the matrix inverse operation in Equation (23) will be invalid. However, the image correlation method does not have this limitation. 

Third, the analytical method needs to associate all the scattering centers correctly before estimating the RV, which is a complex problem in practice. When the scattering centers appear and disappear from the first CPI to the second CPI because of the shadowing effect, there will be some errors after the association of the scattering centers, as shown in Figure 3e. These kinds of errors will have a negative effect on the performance of the analytical method. For the image rotation correlation method, only the strongest scattering center should be associated and moved to the image center. Then, the image rotation is taken around this point. Therefore, the image rotation correlation method outperforms the analytical method when the target has flash points, as proved in the second simulation.

In the end, the image rotation correlation method has a more robust performance when the target has an accelerated rotating movement. Although the RV estimation results with both the above methods deviate from the correct value, as shown in the third simulation, the estimated RV is more reliable with the image correlation method under this situation. Based on these analyses, we can see that the analytical method is very simple and useful when the target rotates with a constant RV, and three or more scattering centers which do not lie on a line can be correctly attracted and associated from the neighboring CPIs. However, the analytical method is just based on the theoretical model, and has some inherent weakness and the image rotation correlation method has a more robust property in practical usage.

## 5. Conclusions

For a given target, narrowband radar should provide larger range coverage than the wideband radar with the same power-aperture product. This paper focuses on the rotating target imaging and parameter estimation with narrow-band radar echoes. The two-dimensional images formed on the velocity-acceleration plane at two neighboring CPIs have been proved to follow a linear mapping relationship. Accordingly, two methods were proposed to estimate the target’s equivalent rotating velocity, which is very important for both the image understanding and target size estimation. The performances of both the rotation estimation methods were provided afterwards, which shows that the rotating velocity may be estimated effectively within a time interval of only a fraction of the rotating period. Experimental results were provided to further demonstrate the robustness of the proposed method when the rotation of the target mismatches the uniformly planar rotation model as exploited in the paper, and the image correlation based rotation estimation method is proved to be more robust to some extent of rotational acceleration. Additionally, the analytic performance of the proposed rotation estimation methods should be investigated in our future work, which may guide the narrowband radar system design for rotational target imaging and feature extraction. What is more, the rotational parameter estimation methods should also be extended to accommodate the accelerated rotation case and even the more complicated three-dimensional rotation situations.
